# Influence of Two Polymer-Based Superplasticizers (Poly-naphthalene Sulfonate, PNS, and Lignosulfonate, LS) on Compressive and Flexural Strength, Freeze-Thaw, and Sulphate Attack Resistance of Lime-Metakaolin Grouts

**DOI:** 10.3390/polym10080824

**Published:** 2018-07-26

**Authors:** Adrián Duran, Jesús F. González-Sánchez, José M. Fernández, Rafael Sirera, Íñigo Navarro-Blasco, José I. Alvarez

**Affiliations:** Heritage, Materials & Environment MIMED Research Group, Departamento de Química, Facultad de Ciencias, Universidad de Navarra, Irunlarrea, 1, 31008 Pamplona, Spain; adrianduran@unav.es (A.D.); jgonzalez.65@alumni.unav.es (J.F.G.-S.); jmfdez@unav.es (J.M.F.); rsirera@unav.es (R.S.); inavarro@unav.es (Í.N.-B.)

**Keywords:** lime-based grouts, metakaolin, polymer-based superplasticizers, freeze-thaw cycles, magnesium sulphate attack

## Abstract

A new range of grouts prepared by air lime and metakaolin (MK) as a pozzolanic admixture has been obtained by using as dispersing agents two polymers, namely poly-naphthalene sulfonate (PNS) and lignosulfonate (LS), with the aim of improving the fluidity of the fresh grouts. Fluidity and setting times of the grouts were assessed. Differences in the molecular architecture and in the anionic charge density explained the different adsorption of the polymers and the different performance. The higher anionic charge of PNS and its linear shape explained its better adsorption and effectiveness. The pozzolanic reaction was favoured in grouts with PNS, achieving the highest values of compressive strength (4.8 MPa after 182 curing days). The addition of PNS on lime grouts slightly decreased the frost resistance of the grouts (from 24 freeze-thaw cycles for the polymer-free samples to 19 or 20 cycles with 0.5 or 1 wt % of PNS). After the magnesium sulphate attack, grouts were altered by decalcification of hydrated phases and by formation of hexahydrite and gypsum. A protective role of portlandite against magnesium sulphate attack was clearly identified. Accordingly, the polymer LS, which preserves a significant amount of Ca(OH)_2_, could be an alternative for the obtaining of grouts requiring high sulphate attack resistance.

## 1. Introduction

Lime-based mortars play an important role in conservation and restoration procedures thanks to their high compatibility with the raw materials employed in the artefacts comprising the Built Heritage [1,2,3]. Grouts based in lime (either air or hydraulic lime) have, in the first approach, an adequate chemical and mechanical compatibility with ancient supports, but may need several additions in order to provide a suitable flowability to fill all the cracks and voids [4,5,6]. In addition, these grouts should fulfil mechanical and durability requirements to guarantee their safe applicability [7,8].

The addition of pozzolanic admixtures is a way of increasing both the final mechanical strength and durability. Specifically, the utilisation of metakaolin (MK) as a pozzolanic addition for mortar and concrete has received extensive attention in the last years. MK is usually processed by calcination of high-purity kaolin clay at temperatures ranging between 650 and 800 °C [9]. It contains silica and alumina in an active form which react with the calcium hydroxide (Ca(OH)_2_, CH) yielding hydrated calcium silicate (C-S-H) phases, and also C_2_ASH_8_ and C_4_AH_13_ as, respectively, hydrated silicoaluminate and hydrated aluminate phases [10,11]. The filler effect of MK and the production of new hydrated phases provide the enhancement of several properties of air-lime based mortars and pastes, such as their setting time or compressive strength, and also reduce microcraking [12].

To provide suitable injectability, polymeric additives (such as superplasticizers, SPs) can be incorporated into the mixture of the fresh grout [12,13]. Superplasticizers enhance the fluidity of the fresh grouts preventing particles from agglomeration, i.e., acting as dispersive agents. In cement-based materials, water-soluble anionic polyelectrolytes, such as polycarboxylate ethers, poly-naphthalene sulfonate (PNS), and lignosulfonate (LS) can be quoted as the most widely used SPs. The chemical structure of the two latter SPs contains hydrophilic (sulfonic groups in both, and also, methoxyl and hydroxyl groups in LS) and hydrophobic parts (naphthalene for PNS and alkylbenzene for LS) [14]. The interaction mechanisms of these polymers are related with the electrostatic and steric forces and also with the adsorption onto surfaces [13,14,15,16,17]. The polymer molecules adsorbed onto binder particles could be able to modify the surface charge (zeta potential) of the particles. Zeta potential values exceeding the range of ±30 mV can lead to electrostatic repulsions between particles avoiding their agglomeration. In addition, electrosteric repulsions between these attached polymer molecules also contribute to the dispersing action [18,19,20]. PNS has been described as a water-reducer agent more efficient than LS [21]. However, LS shows a better plasticizing effect than PNS in some systems [14]. Many works dealt with the effect of these polymers, PNS and LS, in cement systems [16,17,22,23,24,25,26], although there are few articles regarding the performance of these superplasticizers within lime-based mortars [14].

The composition and the relative proportions of each of the components of the grouts affect the fresh as well as the microstructure and mechanical properties of the hardened mortars [2]. This paper focuses on a new range of grouts prepared by air lime, MK as pozzolanic admixture and a polymer-based SP (either PNS or LS). PNS and LS interactions are proposed and fresh and hardened state properties are assessed. The long-term mechanical resistance (compressive and flexural strength) was studied, as well as the durability of the obtained grouts against freeze-thaw cycles and magnesium sulphate attack.

## 2. Materials and Methods

### 2.1. Materials

CL 90-S class slaked lime (ECOBAT Type) in powder form was used for making pastes and grouts. Lime was provided by CALINSA (group Lhoist) (Tiebas, Spain). A limestone aggregate with particle size lower than 2 mm was employed. Aggregate was supplied by CTH (Huarte, Navarra, Spain) and its chemical composition was 52.83% (CaO), 2.28% (MgO), 1.14% (Fe_2_O_3_ + Al_2_O_3_), 0.57% (SO_3_), 0.49% (SiO_2_), 0.07% (Na_2_O), 0.05% (K_2_O), 43.10% (ignition loss). The ratio lime/aggregate was 1:3 by weight. Metakaolin (MK, supplied by METAVER, Pfäffikon, Switzerland) was used as pozzolanic admixture. The MK employed had a specific surface area of 20 m·g^−1^, as measured by the BET method after N_2_ adsorption isotherms (ASAP 2020, Micromeritics, Norcross, GA, USA) and an average particle size of 4.5 µm (particle size distribution determined by laser diffraction in a Malvern Mastersizer, Malvern Instruments, Ltd., Malvern, UK) [13]. Different weight percentages of MK (0, 6, 10, and 20 wt %) with respect to the weight of lime were added.

Two polymers, poly-napthalene sulfonate (PNS) and lignosulfonate (LS) (supplied by FOSROC EUCO S.A., Izurtza, Spain), were assessed as SPs. The characterization of the two polymers focused on the molecular weight, the elemental composition and the anionic charge density of the two polyelectrolytes [11]. The molecular weights, as determined by size-exclusion chromatography (SEC), were 8620 Da for PNS and 8650 Da for LS. Elemental composition (LECO analyser, LECO Corporation, St Joseph, MI, USA) yielded similar values for C (ca. 50%), whereas clear differences were found for sulphur contents: 12.3% for PNS, 6.2% for LS. Titration with Poly-DADMAC allowed obtaining the anionic charge density of the polymers mainly caused by the deprotonation of sulfonate groups. The values, expressed as meq of anionic charge/g of polymer, were 2.44 for PNS and 1.04 for LS, in good agreement with the larger S content determined for PNS.

For testing the properties of the grouts, SPs were added in 0.5 and 1 wt % with respect to the weight of lime. Dosages were selected according to previous values reported in the literature [13,14,27]. To properly assess the effect of the different SPs and their dosages, mixing water was added in a fixed 1:1 water/lime ratio by weight. This ratio of mixing water provided an adequate workability (measured slump in the flow table test within the range 175 ± 5 mm) in the control sample. Table 1 collects the grouts composition.

### 2.2. Experimental Methods

For the preparation of the fresh grouts, lime, metakaolin, and the required amount of SPs (all of them in a dry condition) were blended for 5 min using a solid additives mixer BL-8-CA (Lleal, S.A., Granollers, Spain) to guarantee a proper homogeneity of the components. Mixing water was then added and mixed for 90 s at low speed, in a Proeti ETI 26.0072 (Proeti, Madrid, Spain) mixer. The fluidity of the fresh grouts was measured by using the mini slump flow test according to the norm [28], in which a truncated metallic cone was filled in with the samples and then removed. The slump measurements were recorded after 15 strokes of the flow table, 1 per second, in line with previous works [29]. Density and air content of the fresh grouts were also measured according to the European norms [30,31]. The setting time of the pastes was calculated according to the workable life following the European norm EN 1015-9 [32]. All these experiments were carried out by triplicate and the depicted values are an average value of all the recorded measurements.

Sorption experiments for both SPs (PNS and LS) were carried out following previously referenced processes [13,14,22,33,34] in batch reactors for plain lime pastes (1 g of lime per 25 mL of water) and for lime-MK pastes (5 g of lime and pozzolanic admixture at 6, 10, and 20 wt % with respect to lime in 25 mL of water). The mixtures were stirred for 1 h and, subsequently, centrifuged at 8000× *g* for 15 min. After this, the supernatant was collected and filtered through 0.45 µm PTFE filters. The amount of both SPs adsorbed onto the particles was determined by the difference between the concentration initially added and the final remaining concentration of SP, as quantified by UV–VIS spectrophotometry (maxima at λ = 296 nm for PNS and at λ = 285 nm for LS). The mathematical fitting of the adsorption data was calculated for Langmuir, as well as Freundlich, models.

Regarding the hardened state study, prismatic specimens with dimensions of 160 × 40 × 40 mm were prepared in a Proeti C00901966 mould. The as-prepared grouts were cured at 20 °C and 60% RH and demoulded 7 days later, and stored under those very same conditions that had previously been established for these lime-based mortars [35,36]. Flexural strengths were determined by triplicate in the prismatic specimens using an Ibertest STIB-200 device (Madrid, Spain) at low loading rates of ca. 10 N·m^−1^. Subsequently, compressive strength experiments were executed on the two fragments of each specimen resulting from the flexural tests; the compressive strength experiments were conducted at a rate of loading of ca. 50 N·m^−1^, so that specimens broke between 30 and 90 s. All these tests were carried out according to the European norm [37].

In hardened specimens different characterization methods were performed. For thermal analysis, a simultaneous TG-sDTA 851 Mettler Toledo thermoanalyzer device (Schwerzenbach, Switzerland) was used under the following experimental conditions: alumina crucibles, a temperature range from 25 to 1000 °C, and a heating rate of 10 °C·min^−1^ and static air atmosphere. Fourier transform infrared spectroscopy—attenuated total reflectance (FTIR-ATR) experiments were done in a Shimadzu IRAffinity-1S apparatus (Shimadzu, Japan). The infrared spectra were registered at 100 scans over a wavelength range of 4000–600 cm^−1^, with resolution of 4 cm^−1^. X-ray diffraction (XRD) experiments were performed in a Bruker D8 Advance diffractometer (Bruker, Karslruhe, Germany) with a Cu Kα1 radiation, from 2° to 80° (2θ), 1 s per step, and a step size of 0.04°. A Micromeritics AutoPore IV 9500 apparatus (Micromeritics, Norcross, GA, USA), with a pressure range between 0.0015 and 207 MPa, was used for mercury intrusion porosimetry (MIP) experiments.

For the durability essays, prismatic samples (prepared and cured 28 days as described before) were tested to assess the durability. Hardened grouts were subjected to different processes:

(a) Frost resistance was determined by means of freezing-thawing cycles. The cycles consisted of water immersion of the samples for 24 h and subsequently freezing at −10 °C for 24 h. For these experiments, a CARAVELL 521-102 freezer was used.
(b) Sulphate attack resistance: the monolithic samples were completely submerged in a MgSO_4_ saturated aqueous solution at 20 °C and 95% HR for 24 h. After this process, the samples were dried in an oven at 65 °C for 24 h and submerged in water for 24 h at 20 °C and 95%HR. To conclude the cycle, the specimens were again dried as described above. The cycles were continuously repeated until the total destruction of the specimens.

In order to evaluate the survival of the samples after the ageing cycles, two parameters were considered, following that previously mentioned in other papers [35,36]: (i) compressive strength tests after 7, 14, and 28 cycles when the integrity of the samples allowed them; and (ii) qualitative evaluation based on visual appearance after each cycle; the criterion was the following: degree 0 (samples with no evidence of decay), degree 1 (samples showing a slight degree of deterioration due to some short or thin cracks on surface), degree 2 (altered samples showing some deeper cracks), degree 3 (heavily altered specimens with deep cracks and certain swelling), degree 4 (samples with severe decay due to large and deep cracks and also partial loss or swelling), and degree 5 (completely destroyed samples).

## 3. Results and Discussion

### 3.1. Fresh State Properties

#### 3.1.1. Fluidity, Density, and Air Content

**Figure 1:**
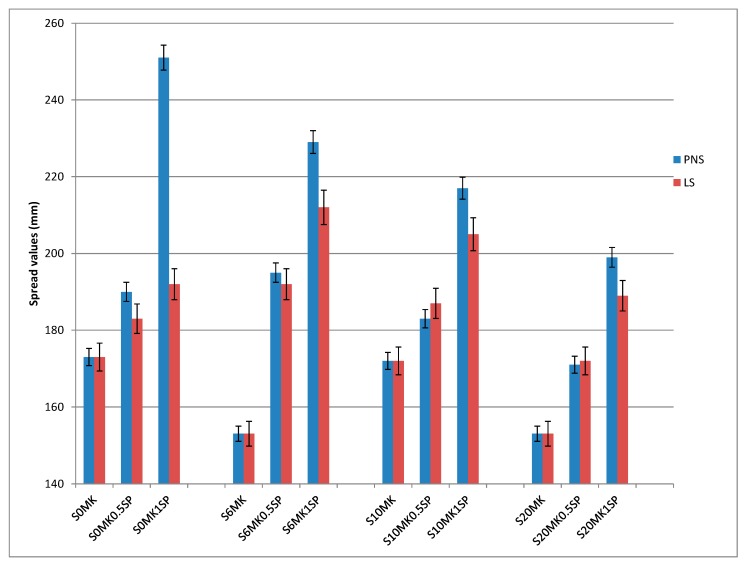
Fluidity values (slump measured in the flow table test) of the different grouts.

Figure 1 shows the fluidity values as a function of the different contents of MK, PNS, and LS in the lime-based grouts (spread values as measured by the flow table test).

The addition of both polymer-based SPs in the plain lime grouts increased the fluidity of the pastes, the incorporation of the PNS being, on average, more effective than that of the LS. The highest dosage of PNS turned out to be the most effective and the two polymers showed a dosage-dependant pattern. The improvement in flowability of the two polymeric dispersive agents supports the interest of the study of these compounds for lime-based grouts.

The presence of MK for polymer-free grouts yielded lower spread values although results did not fit to a dosage-pattern response. In grouts with dispersing polymers, the fluidity was clearly enhanced in the presence of MK. The better efficiency was seen for PNS. The observed results were dissimilar depending on the amount of MK. Two counteracting factors can be taken into account to explain this behaviour: (i) the pozzolanic reaction, giving rise to C-S-H phases and their irreversible agglomeration, resulting in a fluidity decrease; and (ii) the lubricant effect provided by MK that allows the particles to reduce their friction forces, thus increasing the fluidity and workability [4,38]. The increase in MK could lead to an intensification of the pozzolanic reaction, particularly for the highest additions of MK. The absence of a clear trend can be explained considering that the mixing water has been kept constant throughout the work. Samples with high percentages of MK and intensified pozzolanic reaction would require larger mixing water content due to the fast consumption of water. In the case of low amounts of mixing water added, a fluidity decrease would take place. For both superplasticizers, the addition of increasing dosages of MK at the highest SP dosage provoked a reduction in fluidity values. This can be related to the consumption of the polymers during the pozzolanic reaction. Similarly to cement-based materials, the polymers can be adsorbed onto the newly formed hydrates, being then covered by the growing hydration products. These polymer molecules would be unable to act as dispersing agents. The formation of these organo-mineral inactive compounds has been described in the literature [34].

Due to the concurrence of many different factors affecting the fluidity, a careful design of the mix proportions should be considered in order to obtain the most appropriate grouts.

The experimental values of density and air content of the fresh pastes were also determined and collected in Table 2. Although values underwent small variations, there is a consistent slight density reduction as a function of the MK incorporation. This fact can be explained as a consequence of the fixed water/binder ratio, which was kept constant for all the tested grouts. The fast consumption of water on account of the pozzolanic reaction would lead to less-dense packing systems.

Furthermore, the presence of the polymers (PNS and LS) exerted an influence on the density and air-entrained values. LS increased the air-entrained during the mixing process as a result of its surfactant characteristics (with both hydrophobic and hydrophilic segments within the same molecule). Conversely, incorporation of PNS gave rise to lower levels of air content, thus achieving a denser packing system. The excess in the air-entrained together with the low density of the fresh paste could involve a porosity increase after the hardening of the sample, as will be discussed below.

#### 3.1.2. Setting Times

**Figure 2:**
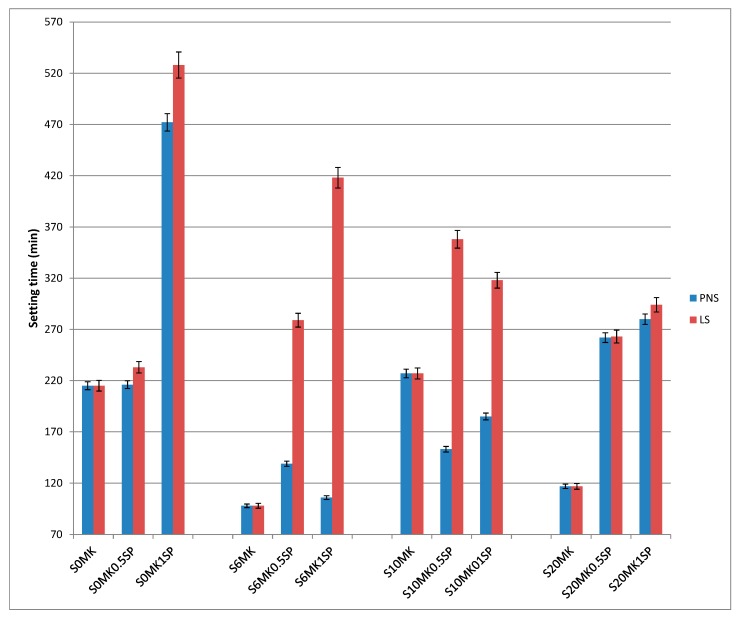
Setting time of the different grouts.

In Figure 2 it can be seen that the addition of LS slowed down the setting time of the fresh grouts. LS caused the strongest delays, especially at the largest dosage tested (Figure 2). The high values of setting times of the binding materials is a frequent inconvenient that arises when using superplasticizers [39,40] and can be due to the interference of the SPs with the growth of the hydration products of the pozzolanic reaction and/or with the carbonation process for pure lime systems.

The setting times did not adjust to a clear pattern. For some grouts, the increasing amount of MK resulted in shorter values of setting times (for example, samples with the largest dosages of LS, Figure 2). This was in line with previous works with other pozzolanic admixtures [14].

For PNS, on average, the increasing amounts of MK, from 6 to 20 wt %, resulted in a setting time delay. The water availability may account on this fact. These fresh grouts were prepared with a fixed water/lime ratio 1:1, obtained from the amount of mixing water required for the control group yielding a consistency of 175 mm (measured in the flow table test). Keeping constant the water/lime ratio and owing to the small particle size of the pozzolanic admixture, the increasing percentages of MK reduced the water availability for the pozzolanic reaction, which should account for a rapid hardening of the fresh mixture.

The main conclusions are that LS caused strong delays in the setting times of lime-MK grouts, whereas PNS was found to be more appropriate when taking into account this parameter. The observed delays for both SPs could reasonably be managed in practical applications.

#### 3.1.3. Adsorption

**Figure 3:**
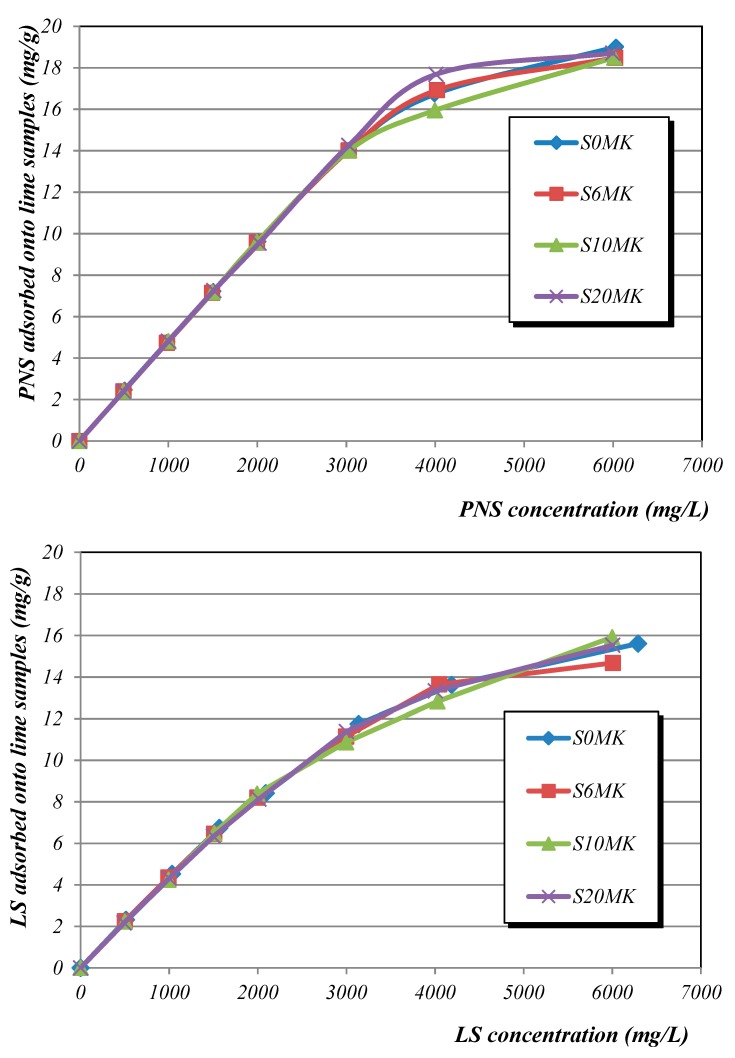
Adsorption isotherms of the SPs onto lime pastes (6, 10, and 20 wt % of pozzolanic admixture). PNS adsorption (**top**); LS adsorption (**bottom**).

Adsorption isotherms were done in order to measure the affinity of PNS and LS for the binder particles in both air lime and MK-air lime media. Particles of air lime and MK-air lime were dispersed in an aqueous media, in which the polymers were then incorporated. After a stirring time, it is expected that some of the polymer molecules had been attached to the particles, whereas some others remain free in the solution, showing a different affinity for the absorbent substrate. Experimental results (Figure 3) showed that PNS was better retained than LS in the tested lime media. In the presence of different percentages of MK only slight differences could be observed for each one of the SPs.

Mathematical treatment of experimental adsorption data have been collected in Table 3. The maximum sorption capacity (q_m_) of PNS was 51.2 mg·g^−1^ for plain lime and 44.7 mg·g^−1^ for lime with 20 wt % of MK. For LS, the maximum sorption capacity value was 32.1 mg·g^−1^ for plain lime and 29.1 mg·g^−1^ for lime with 20 wt % of MK (Table 3). Both SPs showed a better adjustment to a Freundlich model, following a multilayer adsorption model (Table 3) [41,42]. These experimental results, as well as the molecular architecture of the two SPs, can be related to their dispersing effectiveness.

**Figure 4:**
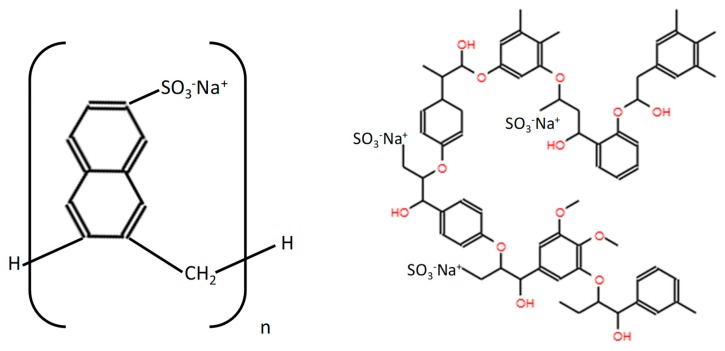
Molecular structure of the two tested polymers: PNS (**left**) and LS (**right**).

The molecular architecture of the LS (Figure 4), with a branchy structure, suggests the steric hindrance as the predominant mechanism. In this work, LS was seen to be less adsorbed [23,43] and to show slightly less plasticizing effects than PNS, which is a linear-shaped SP, but with higher anionic charge density [44,45] (Figure 4). LS has been reported to easily form Ca^2+^ complexes [46,47] and in a previous work the higher ability of LS to bind Ca^2+^ ions has been established [14]. The formation of these LS-Ca^2+^ complexes prevented some LS molecules from being attached to the portlandite, C-S-H, C-S-A-H or C-A-H particles. Furthermore, considering that the anchorage of the polymer onto the active particles takes place by means of favourable electrostatic interaction on the double ionic layer, the higher anionic charge, the more intense the adsorption of the polymer. These facts could explain the lower adsorption of this LS polymer. The adsorption of the polyelectrolyte onto the active particles has been reported to be critical for the dispersing action [47], so that PNS showed higher adsorption and better effectiveness as a dispersing agent in the tested systems.

Finally, the strong Ca^2+^ complexation of the LS would explain its influence on the setting time, preventing lime grouts from carbonation, or even from C-S-H formation.

### 3.2. Hardened State Properties

#### 3.2.1. Mechanical Strength

**Figure 5:**
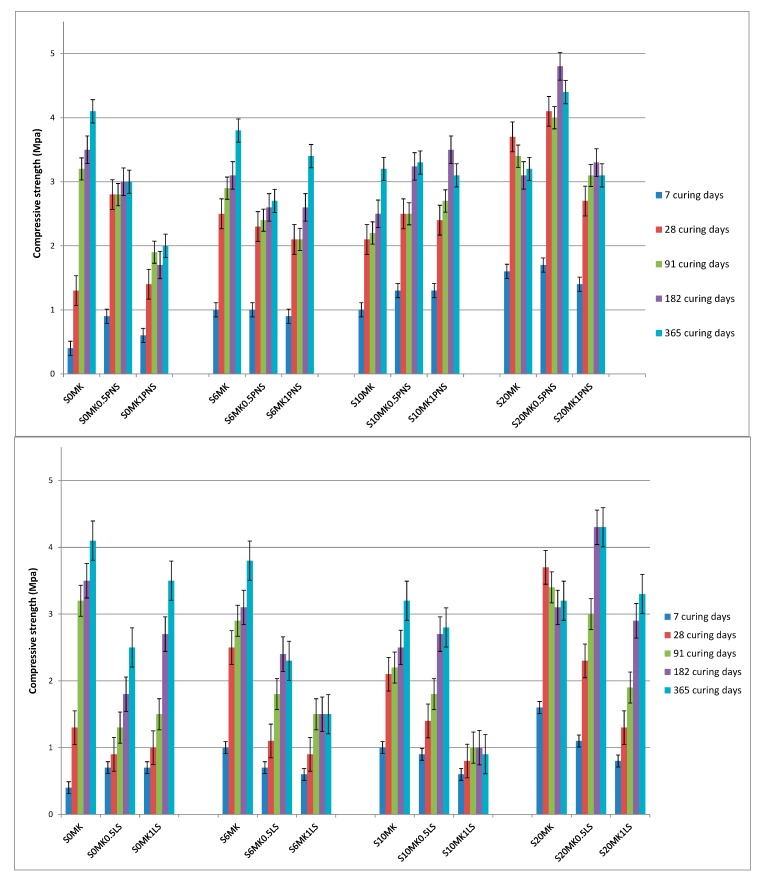
Compressive strength results of the grouts. PNS (**top**); and LS (**bottom**).

Carbonation has a significant influence in the hardening process along time in lime-based systems [48,49]. The mechanical strengths increase over time due to the carbonation process, resulting in the formation of CaCO_3_. Accordingly, on average, the highest values of compressive strength were obtained at long term curing times, usually after one curing year (Figure 5). For plain lime mortars (0% MK), the addition of the SPs caused a drop in the compressive strength values (Figure 5), which can be ascribed to the interference with the lime carbonation process.

The pozzolanic reaction that takes place between CH particles and reactive MK was responsible for the observed mechanical strength improvement at short term in the presence of pozzolanic admixture. This reaction yields C-S-H, C-S-A-H, and C-A-H, according to the data referred in literature [49,50,51] (Figure 5).

The average value of compressive strength was 2.5 MPa for PNS-samples, whereas 1.8 MPa was determined for LS-samples. In the current study, sample S20MK0.5PNS offered the largest values, reaching 4.8 MPa after 182 curing days.

**Figure 6:**
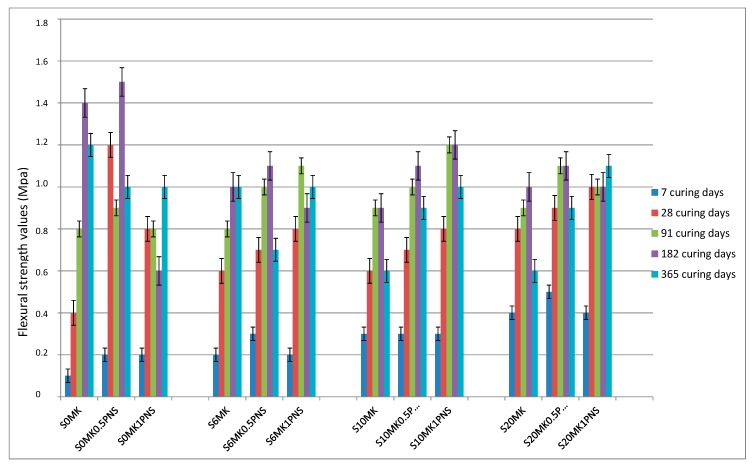

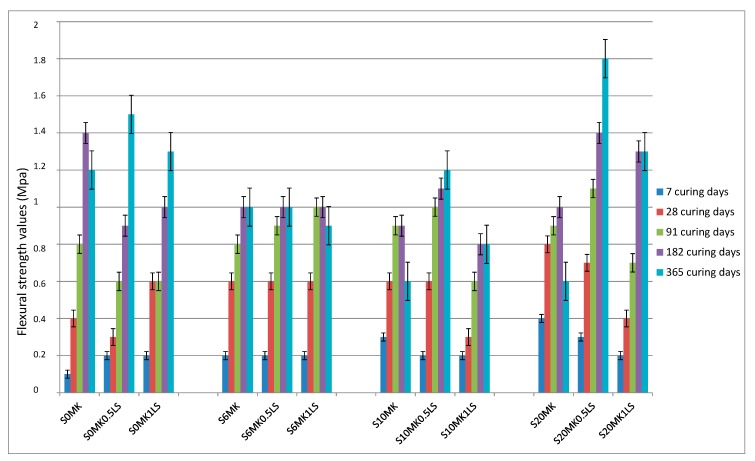
Flexural strength results of the grouts. PNS (**top**); and LS (**bottom**).

Flexural strength values were also measured (Figure 6). The stiffening of the sample due to the C-S-H formation caused a decrease in the flexural strength when MK was incorporated. Polymers were seen to confer different flexural resistance: LS increased the flexural strength, particularly in samples with the highest MK proportions, whereas PNS generally involved a reduction in the flexural strength. Differences in the extent of carbonation and/or pozzolanic reaction could explain these findings, as will be discussed below.

#### 3.2.2. TG-DTA, FTIR-ATR, and XRD Studies

The rate of carbonation and the pozzolanic reaction at the different curing times of the SPs-MK-lime mortars was followed by TG-DTA, FTIR-ATR, and XRD experiments. Previous works also correlated the structure of the materials with the TG measurements [52].

**Figure 7:**
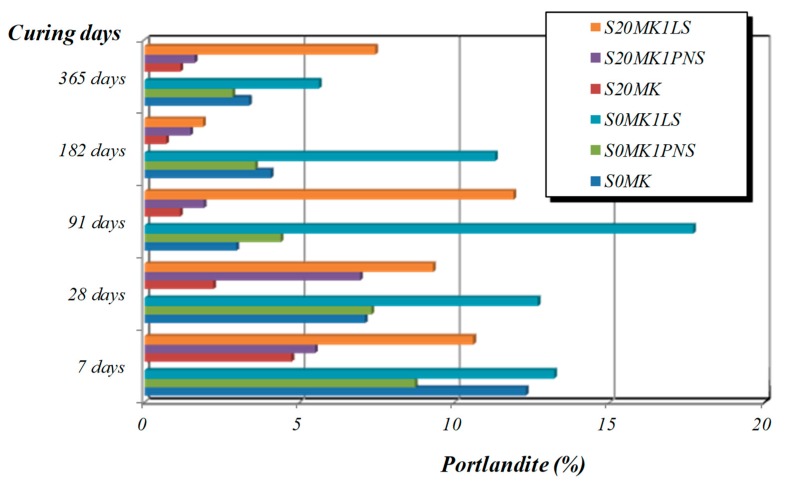
Percentages of Ca(OH)_2_ for mortars at different curing times.

**Figure 8:**
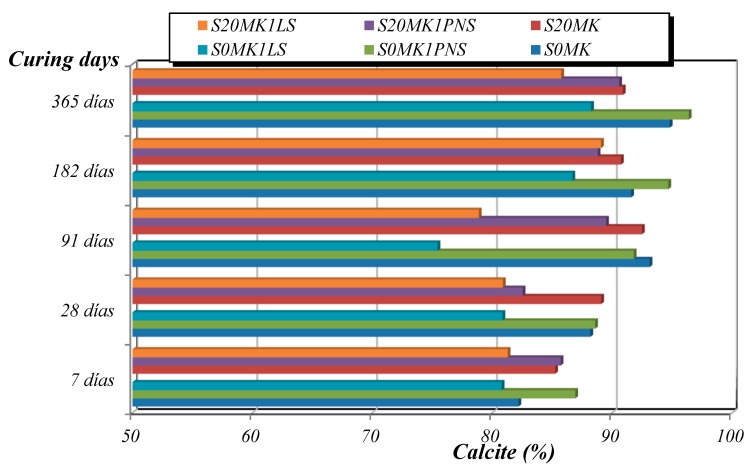
Percentages of CaCO_3_ for mortars at different curing times.

Figure 7 and Figure 8 depict the percentages of Ca(OH)_2_ and CaCO_3_ calculated from TG (weight loss due to dehydroxylation of portlandite at ca. 450 °C, and weight loss owing to the calcite decomposition at ca. 800 °C [53]. The weight loss between 25–300 °C (Table 4) was assigned to the dehydration processes of the calcium silicate (C-S-H), calcium silicoaluminate (C-S-A-H) and calcium aluminate (C-A-H) hydrated phases derived from the pozzolanic reaction, according to some authors [14,54,55,56], and also to residual dehydration of adsorbed water.

For example, the addition of 20% MK (sample S20MK) to the plain lime (sample S0MK) provoked the reduction in the Ca(OH)_2_ content and the increase in the amount of C-S-H, C-A-H and C-S-A-H phases generated by the pozzolanic reaction, as proven by the mass loss increment between 25–300 °C (Table 4). Pozzolanic compounds were identified at the early stages of curing (7 and 28 days) (Table 4), in line with previous results [54]. The highest percentages of CaCO_3_ were found in sample S20MK studied at 91 days and in S0MK and S6MK samples after 365 days. It can be determined that the pozzolanic reaction took place mainly at early stages of curing (7 and 28 days), whereas the carbonation process was significant at longer curing times. This fact could represent a practical advantage in materials used as grouts which will be in contact with water [49,55].

**Figure 9:**
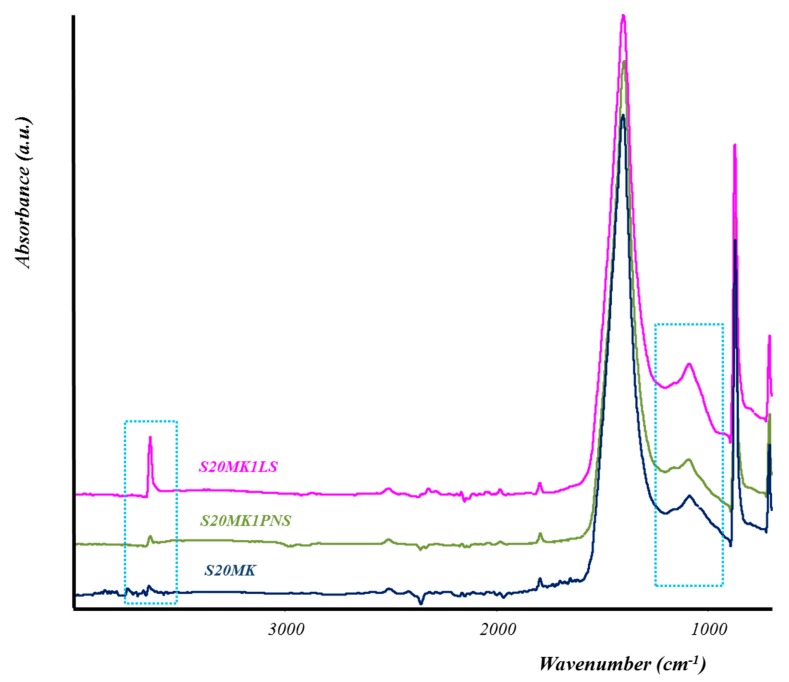
FTIR spectra of different samples after 91 curing days.

Regarding the presence of SPs, the carbonation rate was lower for lime-MK mortars with LS in comparison with samples with PNS. The presence of LS hindered the carbonation process, resulting in higher amounts of unreacted Ca(OH)_2_ and correspondingly lower amounts of CaCO_3_ formed. This was confirmed by the FTIR spectra of lime-based samples containing the highest percentages of LS (1%) and MK (20%) (S20MK1LS). These spectra showed an intense and sharp absorption band at 3600 cm^−1^ ascribed to –OH groups of portlandite, which remained after 91 days (Figure 9, dotted area on the left side of the figure). Conversely, the samples containing PNS (for example S20MK1PNS) did not show the band ascribed to portlandite (Figure 9), due to the higher extent of the carbonation process. Absorption bands at ca. 1400 cm^−1^, 875 cm^−1^, and 712 cm^−1^ were respectively assigned to ν_3_ asymmetric CO_3_ stretching, ν_2_ asymmetric CO_3_ deformation, and ν_4_ symmetric CO_3_ deformation modes [57], and associated to the presence of calcium carbonate (calcite). These results matched with those provided by thermal analysis and were also helpful to justify the compressive strength experiments (values of 1.3 MPa for S20MK1LS and 3.1 MPa for S20MK1PNS at 91 curing days due to a larger carbonation process for the latter).

With respect to the pozzolanic reaction extent, for the higher percentages of MK (20%), the formation of C-S-H, C-S-A-H, and C-A-H compounds, according to the TG values, was also favoured on average for PNS-bearing samples (weight loss of 0.99% for S20MK1PNS sample tested at 28 days and 0.93% at 182 days) in comparison with LS samples (weight loss of.0.70% for S20MK1LS sample tested at 28 days and 0.76% at 182 days) (Table 4). These results explain the higher compressive strengths observed for PNS samples. At the same time, the increase in the stiffening of the sample could result in poorer flexural strengths. The formation of these hydraulic compounds was detected in samples with MK by FTIR measurements. However, spectroscopic results did not offer clear evidence about the comparative rate of formation of hydrated pozzolanic compounds (silicate bands at ca. 1000 cm^−1^, which revealed the presence of C-S-H compounds, the dotted area on the right part of Figure 9) [14,27,35].

In spite of these evidences of the pozzolanic reaction, the identification of the crystalline aluminate and/or silicate phases in the XRD diffractograms was hardly possible. Traces of stratlingite Ca_2_Al(AlSi)O_2_(OH)_10_·2.25H_2_O and cowlesite CaAl_2_Si_3_O_10_.6H_2_O were only detected in samples S20MK and S20MK1PNS (with PNS as SP) (diffraction patterns not shown). Both hydrated compounds have been reported to improve the mechanical strengthening of the samples [54]. The relatively low ratio of pozzolanic admixture, the curing conditions (room temperature and low relative humidity) and the low crystallinity of these hydrated compounds would explain their difficult identification by XRD.

For example, in previous studies larger mass ratios MK:lime were used and curing conditions that favour the pozzolanic reaction (high T/HR) were applied [11,54,58]. In these works, calcium silicate hydrate gel (CSH), stratlingite (C_2_ASH_8_), tetracalcium aluminate hydrate (C_4_AH_13_), monocarboaluminate (C_4_AC^−^CH_11_), katoite (Ca_3_Al_2_(SiO_4_)(OH)_8_), and calcium aluminium hydroxide hydrate (Ca_2_Al(OH)_7_·6.5H_2_O) were identified as phases formed after the lime-MK reaction.

#### 3.2.3. Porosity Measurements

**Figure 10:**
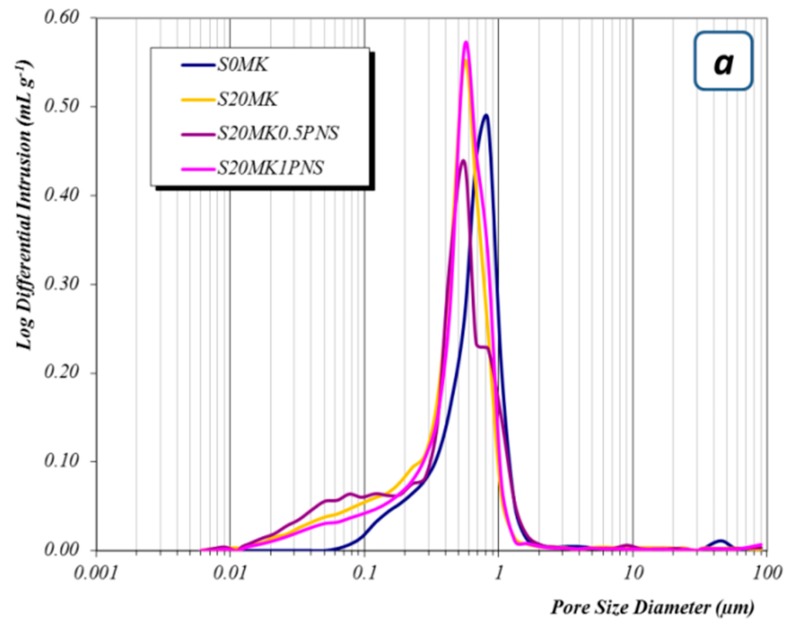

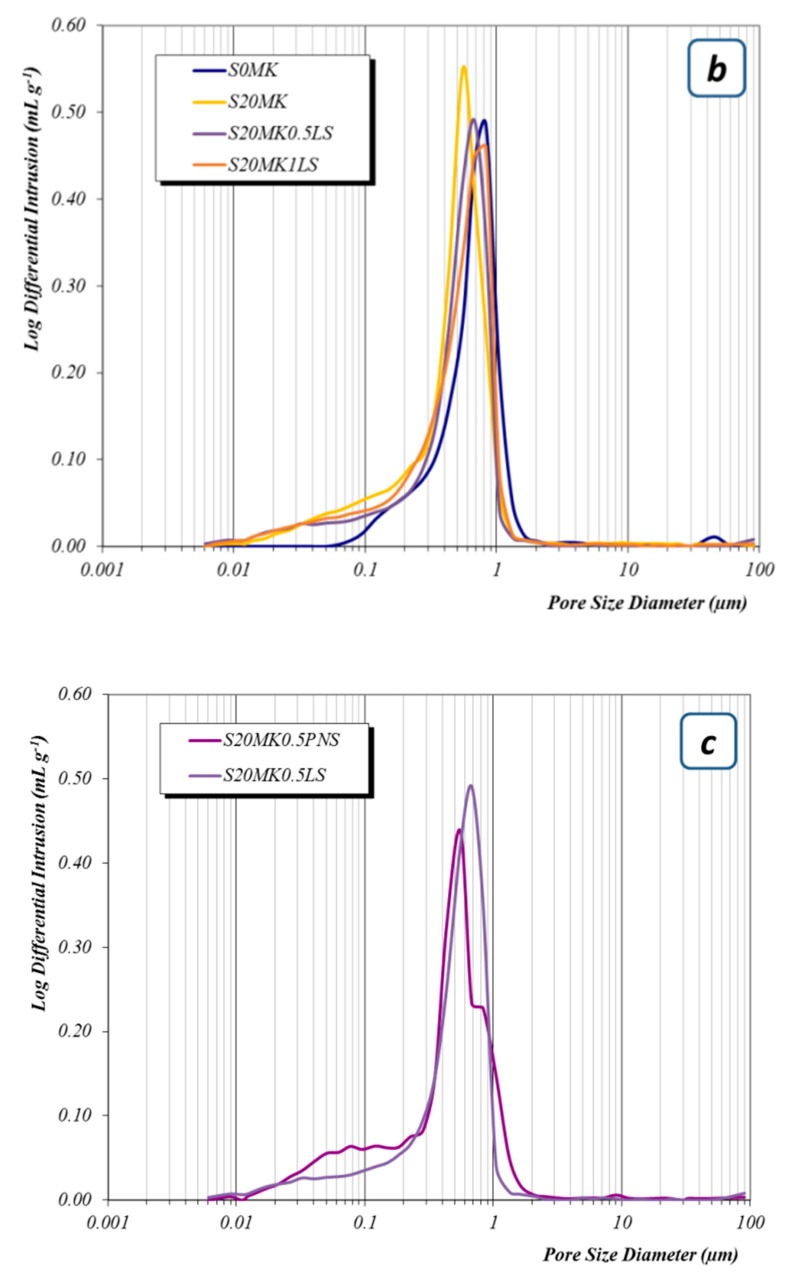
Pore size distribution of different samples tested after 91 curing days. (**a**) control sample, 20 S20MK and samples with PNS, (**b**) control sample, S20MK and samples with LS, (**c**) comparison between samples with 0.5 wt. % of PNS and LS.

The consumption of CH, due to the carbonation progression, and the formation of C-S-H, C-S-A-H, and C-A-H phases gave rise to a refinement of the pore structure, which was studied by mercury intrusion porosimetry (MIP). This technique has been applied in cement-based materials [59]. The addition of MK (S20MK) to the plain lime mortars (S0MK) reduced the mean pore size diameter from 0.83 µm to 0.56 µm in samples tested at 91 curing days (depicted as an example) (Figure 10a). This mean pore size reduction was ascribed to the occurrence of the pozzolanic reaction and also to the filler effect of MK. The filler effect of MK was previously studied, showing that the addition of MK in ordinary Portland cement (OPC) exhibited an important reduction of the permeability and of the porosity when compared with control samples (plain OPC mortars) [60]. In lime mortars this filler effect has also be reported to take place after the incorporation of other pozzolanic compounds, like NS [14,35]. The formation of the new phases by pozzolanic reaction could also contribute to this pore size reduction. These results are in line with the increase in compressive strength observed for MK4 mortars.

The addition of high dosages of PNS to lime mortars did not provoke changes regarding the mean pore size diameter (Figure 10a) but samples showed higher porosity values in the experiments after 91 curing days and, subsequently, lower compressive strength values than those reported for SP-free MK-lime mortars (3.1 MPa for S20MK1PNS vs. 3.4 MPa for S20MK) (Figure 5). The dosage of 0.5% of PNS (S20MK0.5PNS), however, yielded hardened grouts of lower porosity, thus providing higher compressive strength values (4 MPa). The adjustment of the dosage of the SP appears to be imperative to guarantee the mechanical performance of the grouts.

Furthermore, in comparison with LS-bearing grouts (Figure 10b), a higher population of pores in the pore range 0.1–0.01 µm was observed for the grouts containing PNS (Figure 10a). This pore range has been ascribed to the C-S-H pores [61], confirming the extent of the pozzolanic reaction in the presence of PNS.

The incorporation of the LS as a superplasticizer caused an increase in the mean pore size diameter of the grouts (0.56 µm for sample S20MK; 0.68 µm for sample S20MK0.5LS; 0.83 µm for sample S20MK1LS, as depicted in Figure 10b for 91-aged samples). This increase in the pore size explained the compressive strength fall (3.4 MPa for S20MK vs. 3 MPa for S20MK0.5LS vs. 1.3 MPa for sample S20MK1LS) (Figure 5). This fact can be partially related to the air content excess found for this additive in the fresh grouts (Table 2).

The graphical comparison between the pore size distributions of the grouts with both SPs clearly depicts the increment in the main pore size diameter and also in the area under the curve for LS mortars (Figure 10c). At the same time, the smallest diameter pore population related to the C-S-H formation was higher for PNS grouts.

### 3.3. Durability Experiments

#### 3.3.1. Freezing-Thawing

**Figure 11:**
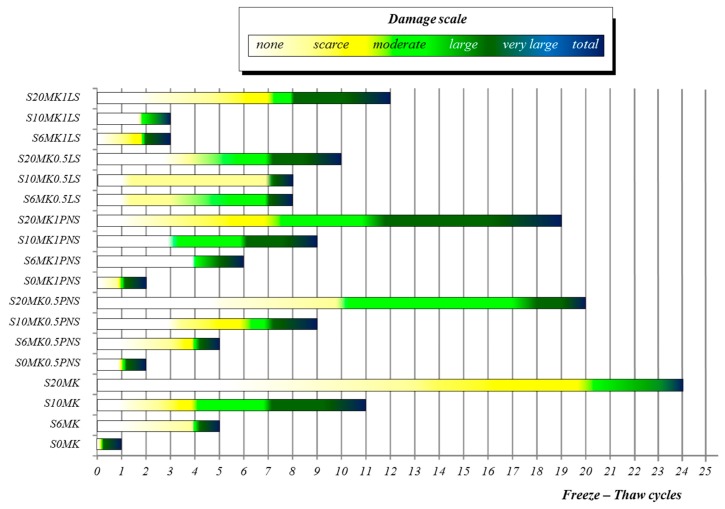
Alteration degrees of grouts after freeze-thaw cycles.

The control group samples subjected to frost resistance test (freezing-thawing F-T cycles) underwent serious decay leading to the total destruction of the sample after just one cycle (Figure 11), in agreement with the poor frost resistance of pure air lime mortars [62]. Fitting itself to a dosage-response pattern, the incorporation of MK clearly enhanced the F-T durability of the grouts. It can be observed that S20MK sample can endure up to 24 F-T cycles displaying serious decays only in the last cycle (Figure 11). The positive F-T endurance provided by the pozzolanic admixture included in lime mortars is in line with the reported incorporation of NS [35]. Nunes and Slizkova [63] assigned this favourable behaviour of mortars comprising of lime + MK to the enhancement of the pozzolanic reaction in wet conditions. Another concomitant factor is the reduction in the mean pore size diameter observed for MK-lime grouts compared with plain lime samples. The decrease in the mean pore size hindered the absorption of liquid water, preventing its later freezing and expansion damage and, consequently, increasing the durability of this type of mortar. Table 5 collects the numerical values corresponding to the different damages observed in the tested samples after 5, 10, 15, and 20 F-T cycles. The numerical value 5 corresponds to the total decay of the specimen and appears marked in red in the Table. Beyond this value, the specimen was totally destroyed and no longer tested.

Compressive strength values (after 7 and 14 F-T cycles) were found to remain appreciable (2.5 and 2.4 MPa, respectively) for grouts with the largest additions of MK (S20MK). In contrast, the flexural strength was significantly affected (values below 0.6 MPa). The fissures observed on S20MK mainly appeared on the side faces. The compressive strength is parallel to the longitudinal cracks so it is unaffected. However, the flexural strength is significantly affected by the cracks [63].

**Figure 12:**
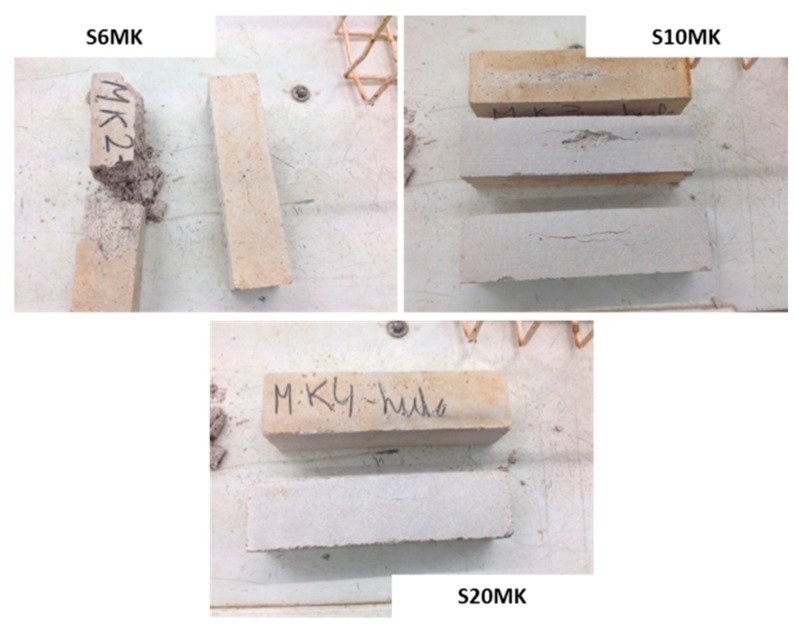

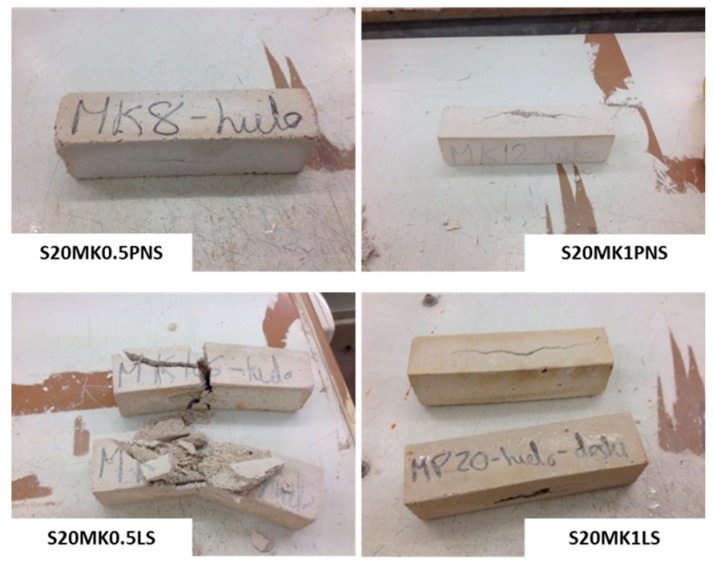
Appearance of different grouts after 10 freeze-thaw cycles.

The addition of PNS in the MK-air lime was only slightly detrimental for the durability of the samples, i.e., S20MK1PNS grouts suffered total decay after 19 F-T cycles, and S20MK0.5PNS (with lower porosity) after 20 cycles. Figure 12 showed images of three hardened grouts, S20MK, S20MK0.5PNS, and S20MK1PNS, after 10 F-T cycles. The fissures are clearly visible in S20MK0.5PNS and in S20MK1PNS.

Conversely, the use of LS significantly harmed the F-T durability of the grouts, with total decay after only 10 and 12 F-T cycles, respectively, for S20MK0.5LS and S20MK1LS grouts. The highest increase in mean pore size of the LS-bearing samples, providing a higher absorption of liquid water during the durability test, may contribute to clarifying this experimental finding.

In order to visually compare the durability performance with both SPs, Figure 12 depicted images corresponding to samples S20MK0.5PNS, S20MK0.5LS, S20MK1PNS, and S20MK1LS after 10 F-T cycles. As mentioned before, at that stage, the highest decay was found for S20MK0.5LS. Important deterioration was also observed for S20MK1LS and S20MK1PNS, in which surface fissures were obvious.

#### 3.3.2. Magnesium Sulphate Attack

**Figure 13:**
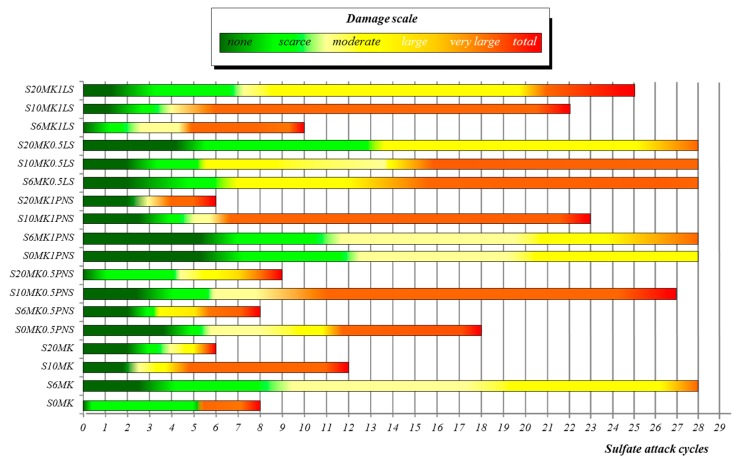
Alteration degrees of grouts after sulphate attack cycles.

The assessment of the resistance of sulphate attack of MgSO_4_ was also carried out. Results are gathered in Figure 13. Table 6 displays the numerical values corresponding to the different damages observed in the tested samples after 5, 10, 15, 20 and 25 sulphate attack cycles. The numerical value 5 corresponds to the total decay of the specimen and appears marked in red in the Table. Beyond this value, the specimen was totally destroyed and no longer tested.

Opposite to F-T resistance, the increase in wt % MK addition damage the sulphate attack resistance of the grouts. Whilst samples with 6 wt % MK (S6MK) lasted 27 cycles with an intermediate alteration degree (degree 3), samples with 10 wt %MK (S10MK) only lasted 12 cycles before reaching a complete decay, and samples with 20 wt % (S20MK) reached a total decay after 6 cycles.

**Figure 14:**
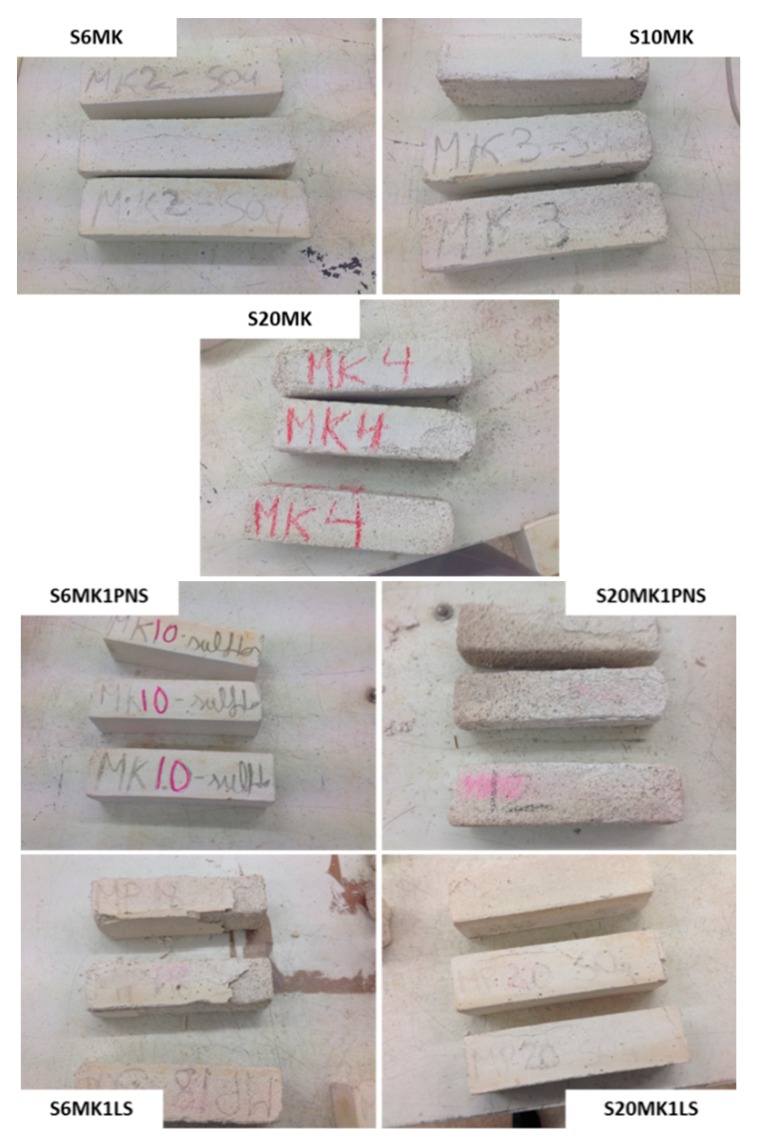
Appearance of different grouts after five sulphate crystallization cycles.

In this sense, Figure 14 showed images of S6MK, S10MK and S20MK grouts after 5 sulphate cycles. In the sample S20MK, the spalling of the superficial part of the specimens and partial disintegration took place [35]. This result suggested the presence of sulphate compounds at the surfaces [35,62,63,64].

The PNS addition in a 0.5% dosage yielded the highest tolerance to the sulphate attack for S10MK0.5PNS. When the percentage of PNS was increased to 1%, the highest endurance was found for the lower percentage of MK (S6MK1PNS). In the case of the addition of LS on MK-lime mortars, a linear behaviour was observed: the larger the amount of MK, the higher the durability against sulphate attack cycles. To illustrate these results, Figure 14 depicts images corresponding to samples S6MK1PNS, S6MK1LS, S20MK1PNS, S20MK1LS after 5 sulphate attack cycles. Severe decays and losses of a part of the mortars were observed for grouts S20MK1PNS and S6MK1LS at this stage.

In the same line of the detrimental presence of MK, owing to the enhancement of C-S-H, C-S-A-H, and C-A-H phases when PNS was present, PNS-bearing grouts showed a worse sulphate attack resistance. The literature has shown that Mg^2+^ ions (in case of magnesium sulphate attack) cause decalcification of C-S-H, increasing the degree of alteration [65]. This is in line with the observed stronger damage in samples S20MK and S20MK1PNS, which presented a large amount of C-S-H, as discussed before.

A detailed examination by XRD of the grouts after three cycles of the sulphate attack revealed the formation of expansive hydrated compounds and soluble salts, such as hexahydrite and gypsum (MgSO_4_·6H_2_O and CaSO_4_·2H_2_O). These compounds were responsible for the degradation of the samples. Furthermore, it was seen that the lower the amount of uncarbonated portlandite, the stronger the formation of degradation salts. The presence of portlandite hindered the formation of these sulphates, possibly by the precipitation of magnesium hydroxide (brucite) that was also detected in some of the XRD patterns.

A quantitative phase analysis was carried out by means of a Rietveld refinement of the XRD patterns with TOPAS software. As an example, comparative percentages of the samples with 20% MK are collected in Table 7.

These results are in complete agreement with the presence of portlandite reported in Figure 7. Samples with the lowest percentages of portlandite (S20MK and S20MK1PNS) showed the highest percentages of expansive and soluble sulphate salts. The grout with the highest percentage of portlandite (S20MK1LS) yielded the lower amount of expansive hexahydrite. At the same time, this grout showed the highest percentage of brucite, confirming the protective role of the portlandite, which trapped Mg^2+^ ions delaying their decay activity [66,67].

Although in previous works the growth of expansive phases, such as ettringite and thaumasite, had been reported to constitute an important mechanism of degradation [64] of cementitious samples subjected to sulphate attack, in this work there was no evidence of the formation of these compounds in the tested grouts.

Two different mechanisms took place concerning the durability of the tested grouts. The refinement of the pore structure caused by the presence of MK and PNS (filler effect and pozzolanic reaction) enhanced the frost resistance of the mortars by hindering the water access. However, the appearance of C-S-H, C-A-H, and C-S-A-H impaired the sulphate attack resistance that caused decalcification of these phases. The presence of Ca(OH)_2_ had a protective effect delaying the decay induced by Mg^2+^ by precipitation of Mg(OH)_2_. The sulphate attack was seen to be strongly dependent of the chemical and mineralogical composition of the grouts [64,68].

## 4. Conclusions

The fluidity of the lime grouts that also contained MK as a pozzolanic admixture was clearly increased upon the addition of the two tested polymer-based superplasticizers (LS and PNS). Among the two tested polymers, PNS showed higher dispersing effect than LS on account of its higher adsorption onto portlandite, C-S-H, C-S-A-H, and C-A-H particles. The higher anionic charge of the polyelectrolyte PNS and its linear molecular architecture explained its better adsorption. Setting times were less affected for PNS addition than for LS incorporation. LS was seen to cause delays in the setting time.

The pozzolanic reaction was favoured in grouts with PNS, consequently the highest values of compressive strength were reached when this polymer was employed, i.e., 4.8 MPa after 182 days in samples with 20% MK and 0.5% PNS.

The incorporation of MK enhanced the freezing-thawing durability of the grouts due to the decrease in the mean pore size that consequently hampered the absorption of liquid water and reduced the damage by freezing and expansion of the retained water in pores. The addition of PNS on lime grouts slightly decreased the F-T durability of the grouts (from enduring 24 F-T (0% PNS) to 19 F-T or 20 F-T, with 0.5 and 1 wt % of PNS, respectively), so that the enhancement in fluidity, compressive strength, and frost resistance provided by this polymer supports its use for lime-based grouts.

However, the formation of C-S-H, C-S-A-H, and C-A-H was preferred in the presence of PNS as polymer, and the appearance of these phases results in a weaker resistance against sulphate attack. Grouts were altered by decalcification of hydrated phases and by formation of hexahydrite and gypsum. A protective role of portlandite against magnesium sulphate attack was clearly identified. Accordingly, the polymer LS, which preserve a significant amount of Ca(OH)_2_, could be an alternative for the obtaining of grouts requiring high sulphate attack resistance.

## Figures and Tables

**Table 1:** Composition of the different grouts (all of them were prepared with 500 g of air lime, 1500 g of calcitic sand, and 500 g of mixing water).

Samples	MK (g)	LS (g)	PNS (g)
S0MK (control group)	0	0	0
S0MK0.5LS	0	2.5	0
S0MK0.5PNS	0	0	2.5
S0MK1LS	0	5	0
S0MK1PNS	0	0	5
S6MK	30	0	0
S6MK0.5LS	30	2.5	0
S6MK0.5PNS	30	0	2.5
S6MK1LS	30	5	0
S6MK1PNS	30	0	5
S10MK	50	0	0
S10MK0.5LS	50	2.5	0
S10MK0.5PNS	50	0	2.5
S10MK1LS	50	5	0
S10MK1PNS	50	0	5
S20MK	100	0	0
S20MK0.5LS	100	2.5	0
S20MK0.5PNS	100	0	2.5
S20MK1LS	100	5	0
S20MK1PNS	100	0	5

MK: metakaolin; LS: lignosulfonate; PNS: poly-naphthalene sulfonate.

**Table 2:** Bulk density and air content of the fresh grouts.

Samples	Bulk Density of the Fresh Paste (g·mL^−1^)	Air Content (%)
S0MK (control group)	1.89	3.2
S0MK0.5LS	1.90	3.4
S0MK0.5PNS	1.90	2.6
S0MK1LS	1.89	3.4
S0MK1PNS	1.93	1.6
S6MK	1.89	3.0
S6MK0.5LS	1.89	3.2
S6MK0.5PNS	1.88	3.0
S6MK1LS	1.89	3.3
S6MK1PNS	1.90	2.2
S10MK	1.89	3.3
S10MK0.5LS	1.89	3.2
S10MK0.5PNS	1.89	2.7
S10MK1LS	1.88	3.4
S10MK1PNS	1.89	2.5
S20MK	1.87	3.1
S20MK0.5LS	1.87	3.4
S20MK0.5PNS	1.89	3.0
S20MK1LS	1.87	3.2
S20MK1PNS	1.89	3.0

**Table 3:** Results of adsorption isotherms onto air lime suspensions at different MK percentages: Langmuir and Freundlich adsorption parameters for both SPs.

**Polynaphthalene Sulfonate (PNS)**						
	Langmuir			Freundlich		
	q_m_ (mg·g^−1^)	b	R^2^	K	1/n	R^2^
S0MK	51.2	0.00011	0.7738	0.01210	0.8658	0.9757
S6MK	46.9	0.00011	0.8019	0.01215	0.8582	0.9768
S10MK	43.4	0.00012	0.8469	0.01247	0.8490	0.9775
S20MK	44.7	0.00010	0.7458	0.00986	0.8708	0.9766
**Lignosulfonate (LS)**						
	Langmuir			Freundlich		
	q_m_ (mg·g^−1^)	b	R^2^	K	1/n	R^2^
S0MK	32.1	0.00016	0.9509	0.01983	0.7812	0.9775
S6MK	28.7	0.00018	0.9242	0.01837	0.7830	0.9735
S10MK	31.5	0.00015	0.9647	0.01582	0.7977	0.9825
S20MK	29.1	0.00015	0.9400	0.01346	0.8076	0.9809

**Table 4:** TG results of weight loss between 25–300 °C, assigned to dehydration of pozzolanic compounds.

Samples	Weight Loss (%)				
	7 Days	28 Days	91 Days	182 Days	365 Days
S0MK (Plain lime)	0.38	0.74	0.45	0.30	0.31
S20MK	1.03	0.88	0.57	0.73	0.66
S20MK1PNS	0.76	1.00	0.75	0.93	0.58
S20MK1LS	0.51	0.70	0.61	0.76	0.80

**Table 5:** Visual alteration after 5, 10, 15, and 20 freezing-thawing (FT) cycles showing numerical values of the damage scale *.

	Number of FT Cycles			
Samples	5	10	15	20
S20MK1LS	2	4	**5**	-
S10MK1LS	**5**	-	-	-
S6MK1LS	**5**	-	-	-
S20MK0.5LS	3	**5**	-	-
S10MK0.5LS	3	**5**	-	-
S6MK0.5LS	4	**5**	-	-
S20MK1PNS	2	3	4	**5**
S10MK1PNS	3	**5**	-	-
S6MK1PNS	4	**5**	-	-
S20MK0.5PNS	0	2	4	**5**
S10MK0.5PNS	3	**5**	-	-
S6MK0.5PNS	**5**	-	-	-
S0MK0.5PNS	**5**	-	-	-
S20MK	0	1	2	3
S10MK	3	4	**5**	-
S6MK	**5**	-	-	-
S0MK (control group)	**5**	-	-	-

* Damage scale: 0: none; 1: scarce; 2: moderate; 3: large; 4: very large, 5 (in red): total decay of the specimen.

**Table 6:** Visual alteration after 5, 10, 15, 20, and 25 magnesium sulphate attack cycles, showing the numerical values of the damage scale *.

	Number of Sulphate Attack Cycles				
Samples	5	10	15	20	25
S20MK1LS	1	3	3	4	**5**
S10MK1LS	4	4	4	**5**	-
S6MK1LS	4	**5**	-	-	-
S20MK0.5LS	0	1	2	3	3
S10MK0.5LS	1	2	4	4	4
S6MK0.5LS	1	2	4	4	4
S20MK1PNS	4	**5**	-	-	-
S10MK1PNS	1	4	4	**5**	-
S6MK1PNS	0	1	2	3	4
S20MK0.5PNS	2	**5**	-	-	-
S10MK0.5PNS	1	2	4	4	**5**
S6MK0.5PNS	3	**5**	-	-	-
S0MK0.5PNS	0	2	4	**5**	-
S20MK	3	**5**	-	-	-
S10MK	4	4	**5**	-	-
S6MK	1	2	2	3	3
S0MK (control group)	1	**5**	-	-	-

* Damage scale: 0: none; 1: scarce; 2: moderate; 3: large; 4: very large, 5 (in red): total decay of the specimen.

**Table 7:** Results of the Rietveld quantitative phase analysis of the XRD after three sulphate attack cycles, showing the percentages of the different phases.

Samples	Phase (wt %)					
	Calcite	Portlandite	Brucite	Quartz	Gypsum	Hexahydrite
*S20MK*	86.2	-	-	0.7	5.6	7.5
*S20MK1PNS*	82.6	0.4	1.0	0.4	10.3	5.3
*S20MK1LS*	81.1	4.3	3.0	0.4	9.1	2.1
